# Risk factors of pressure injury in elderly inpatients: a systematic review and meta-analysis

**DOI:** 10.1186/s12877-025-06517-0

**Published:** 2025-11-10

**Authors:** Qingyi Wu, Nini Cheng, Fanfan Cao, Huan Wen, Mei Sun

**Affiliations:** 1 Xiangya School of Nursing, Central South University, Changsha, China; 2 Department of Urology, The First Affiliated Hospital of Xiamen University, Xiamen, China

**Keywords:** Elderly, Inpatients, Pressure injury, Risk factors

## Abstract

The aim of this systematic review is to systematically identify and synthesize the risk factors contributing to pressure injury in elderly inpatients. Methods: PubMed, Embase, Web of Science, Cochrane Library, and CINAHL were systematically searched from database inception to March 1, 2025. Two researchers independently screened the retrieved studies and extracted the data. The risk of bias was assessed using the AHRQ criteria or the Newcastle–Ottawa Scale (NOS). Meta-analysis was performed using R version 4.5.0. Results: A total of 3,629 studies were retrieved, and 23 studies met the inclusion criteria. Altogether, 70,340 participants were included in the analysis, of whom 5,792 developed PI during hospitalization, corresponding to an overall prevalence of 8.2%. Meta-analysis showed that age (*OR* = 1.06, 95%*CI*: 1.03 to 1.09), immobility (*OR* = 4.54, 95%*CI*: 3.09 to 6.67), incontinence (*OR* = 4.54, 95%*CI*: 2.33 to 42.75), nutritional risk (*OR* = 3.00, 95%*CI*: 1.78 to 5.05), diabetes (*OR* = 1.60, 95%*CI*: 1.21 to 2.11), durations of prior ICU stay (*OR* = 1.93, 95%*CI*: 1.46 to 2.55), time from admission to surgery (*OR* = 2.07, 95%*CI*: 1.60 to 2.67) and length of stay (*OR* = 1.05, 95%*CI*: 1.01 to 1.01) were associated with pressure injury in elderly inpatients. Conclusions: Pressure injury remains a serious and preventable complication among elderly inpatients. This study provided a comprehensive evaluation of the risk factors of pressure injury development among elderly inpatients, identifying eight major contributors. These findings may promote valuable evidence to inform clinical assessment, risk stratification, and the development of targeted prevention strategies.

**Supplementary Information:**

The online version contains supplementary material available at 10.1186/s12877-025-06517-0.

## Introduction

Pressure injury (PI) is defined as localized damage to the skin and/or underlying tissue, usually over a bony prominence or associated with medical or other devices, resulting from prolonged pressure or pressure in combination with shear [1]. PI is a significant clinical problem that requires urgent attention, especially among older adults. On the one hand, epidemiological evidence shows that the prevalence of PI remains high in hospital settings worldwide, with older adults being disproportionately affected. A global systematic review and meta-analysis [2] involving including more than 2.5 million hospitalized adult patients reported a pooled PI prevalence of 12.8%, with hospital-acquired pressure injury rates accounting for nearly two-thirds of all cases; another global burden of disease study [3] revealed that the age-standardized prevalence rates of PI were notably higher in individuals aged 60 and older compared to younger age groups. On the other hand, PI has sever negative impacts on patients’ physical functioning, mental health, and social participation [4], even leading to death/medical malpractice [5]. Furthermore, PI also imposes a substantial burden on society, leading to considerable healthcare and treatment costs. In Australia, the total cost of PI in public hospitals was estimated at $9.11 billion, including $3.60 billion attributable to the opportunity cost of excess length of stay and $3.59 billion to treatment expenses [6]. Therefore, the high prevalence, severe consequences, and heavy economic burden make PI a major health challenge for elderly patients, healthcare systems, and society.

The aging population bringing numerous health challenges, particularly regarding PI. According to the World Health Organization, the global population aged 60 years and older is projected to reach 2.1 billion by 2050, nearly doubling from 1 billion in 2020 [7]. This demographic shift suggests that the number of elderly patients at risk of PI will increase significantly. Therefore, it is essential to identify risk factors for PI in elderly inpatients and implement targeted preventive measures. However, most existing studies have focused on individual factors, and a critical gap remains: no comprehensive systematic evaluation has synthesized all known risk factors to clearly delineate their relationships with PI development in elderly inpatients. This study systematically reviewed the literature on the risk factors of PI in elderly hospitalized patients and conducted a Meta-analysis on the risk factors. Addressing the gap may enable clinicians to better stratify risk, prioritize interventions, and develop evidence-based prevention protocols tailored to elderly inpatients.

## Method

The study was conducted in accordance with the Preferred Reporting Items for Systematic Reviews and Meta-Analyses (PRISMA) guidelines[8], and the protocol has been registered on PROSPERO (CRD420251010457).

### Search strategy

The studies search was performed in PubMed, Embase, Web of Science, Cochrane Library, and CINAHL from inception to March 1, 2025. Search terms were “Older Adults”, “Inpatients”, “Pressure Ulcer” and “Risk Factors”. We used a combination of subject terms and free words, Boolean logical operators, and truncation to search in the target database. The full search strategy can be found in Appendix 1.

### Eligibility criteria

Inclusion Criteria: We included cohort, case-control and cross-sectional studies that investigated elderly inpatients, defined as individuals aged 60 years or older who are admitted to a hospital for any forms of inpatient care; Eligible studies examined risk factors associated with PI, such as nutritional status[9], level of physical activity[10], chronic disease[11], urinary incontinence [12] or length of hospitalization[13], and reported outcomes of PI during hospitalization [14]. Only articles published in English were considered.

Exclusion Criteria: We excluded studies that were not original research (e.g., reviews, editorials, conference abstracts), those involving residents of nursing homes or long-term care facilities, and studies in which PI had already occurred at the time of hospital admission.

### Screening and data extraction

Two researchers independently screened the retrieved studies and extracted data using EndNote21, following Eligibility Criteria. The results were cross-checked for accuracy. Any disagreements were resolved through discussion, and when consensus could not be reached, the second author served as the third researcher for adjudication. Extracts include: first author, year of publication, country, sample size, age (mean ± standard deviation or range), risk factors with *P* < 0.05 and their effect values and 95% confidence intervals (CI).

### Quality assessment

Cross-sectional studies were assessed using the Agency for Healthcare Research and Quality (AHRQ) [15] criteria, which includes 11 items. Studies scoring 8 to 11 were considered high quality, 4 to 7 were considered medium quality, and 0 to 3 were considered low quality. Case-control and cohort studies were evaluated using the Newcastle-Ottawa Scale (NOS) [16]. The evaluation consisted of 3 columns with 8 entries, which were scored according to the criteria of the entries. 5 to 9 were categorized as high quality, and 0 to 4 were categorized as low quality.

### Data analysis

All statistical analyses were conducted using the R software (version 4.5.0), specifically employing the “metafor”[17] package. The natural logarithms of the adjusted odds ratios (*aOR*) and 95%*CI* were used to calculate effect estimates and corresponding standard errors (*SEs*). Within-study variances were computed as the squared *SEs*. One study reported the association as a regression coefficient (*β*) from a logistic regression model. This value was transformed into an *aOR* using exponentiation (*aOR* = exp[*β*]) to enable its inclusion in the meta-analysis. Additionally, effect estimates such as risk ratio (*RR*), adjusted risk ratio (*aRR*), and adjusted hazard ratio (*aHR*) reported by only one study, respectively, were excluded from meta-analysis. They were instead presented narratively to complement the quantitative findings.

A random-effects model was primarily used to account for potential heterogeneity among studies. Heterogeneity among studies was assessed using the *I²*, *τ²*, and Cochran’s Q test. *I²* value between 0% and 40% might not be important, 30% to 60% may indicate moderate heterogeneity, 50% to 90% may represent substantial heterogeneity, and 75% to 100% suggests considerable heterogeneity [18]. If heterogeneity is low, the results of the fixed effects model are reported.

Sensitivity analyses were conducted by comparing results from fixed-effects and random-effects models. Forest plots were used to visually present individual and pooled effect estimates. A significance level of *P* < 0.05 was considered statistically significant.

## Results

### Characteristics of included studies

**Fig. 1:**
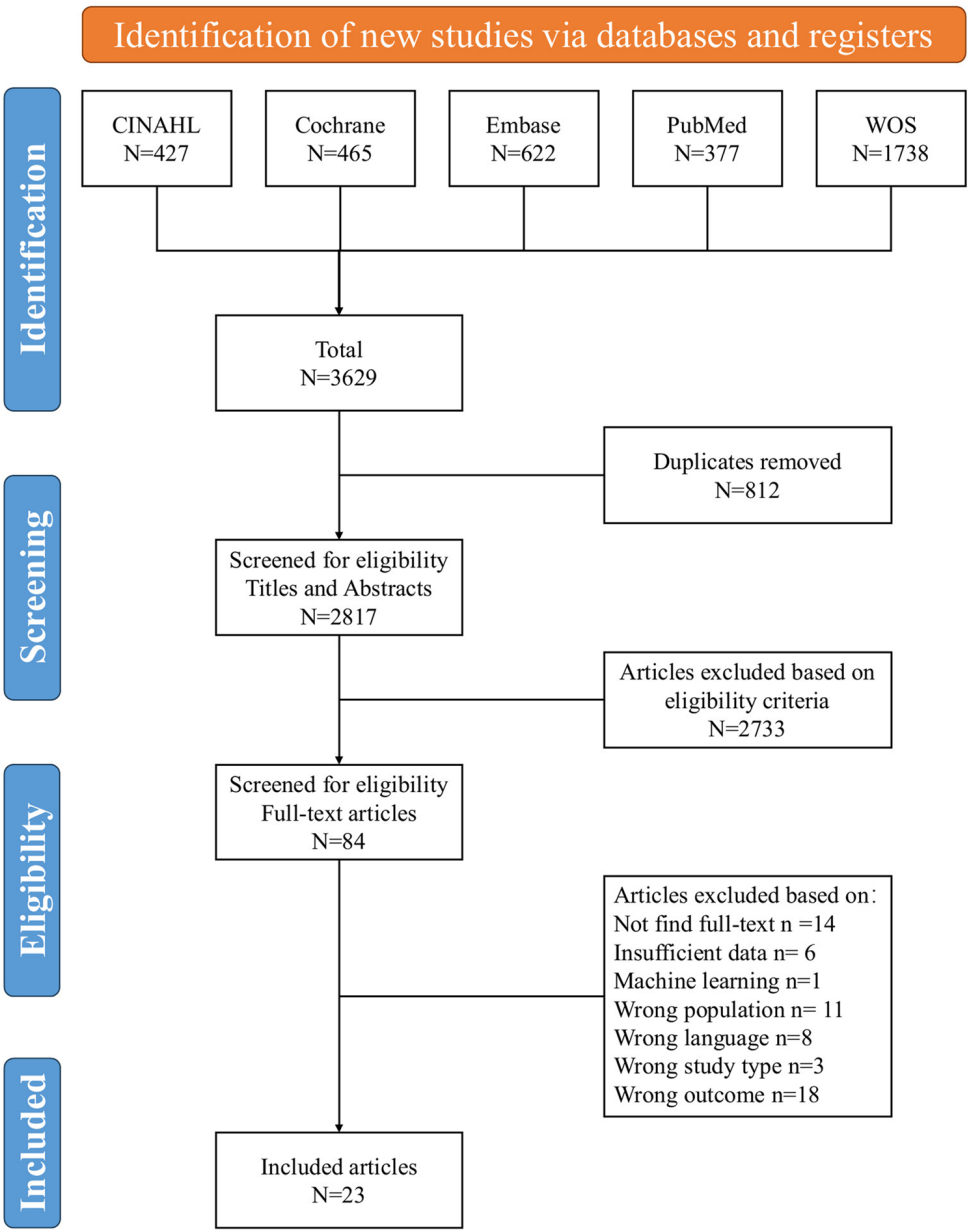
Flowchart of section studies

A total of 3629 studies were retrieved 2817 remaining after eliminating duplicates, and finally 23 studies[9–13, 19–36] met eligibility criteria, including 1 case-control study, 4 cross-sectional studies, and 18 retrospective cohort studies. The process of studies screening is shown in Fig. 1. Detailed information on the literature characteristics for each included study can be found in Table 1.

**Table 1:** Basic characteristics of included studies

Author	Year	Country	Design	Case/Total	Age	Risk Factors	Effect values	Scores
Anthony	2000	England	Cohort	113/773	≥ 65	Waterlow score, albumin	*aOR*	8
Baumgarten	2003	USA	Cohort	824/9400	≥ 60	time to surgery, surgical anesthesia, durations of prior ICU stay, age, ADL, nutritional risk, health status bad, Charlson Comorbidity Index	*aOR*	9
Baumgarten	2006	USA	Cohort	266/3233	≥ 65	age, male, dry skin, incontinence, immobility, nursing home, hospitalized history, nutritional risk	*aOR*	7
Baumgarten	2008	USA	Case-control	195/792	≥ 65	durations of prior ICU stay, medication	*aOR*	8
Baumgarten	2012	USA	Cohort	96/658	83.2 ± 6.6	length of stay in emergency department, time from inpatient admission to surgery, general anesthesia	*aRR*	8
Casimiro	2002	Spain	Cross-sectional	295/827	82.4 ± 8.0	age, BMI, history of ulcer, immobility, poor circulation, diabetes, sensory changes, erythema	*aOR*	8
Cataneo-Piña	2023	Mexico	Cohort	37/118	82.1 ± 5.6	length of stay	*aOR*	8
Chiari	2017	Italy	Cohort	246/1083	84.1 ± 7.6	age, railings on the bed, positioning, catheter, caregiver, foam valve, pain	*aOR*	8
Compher	2007	USA	Cohort	608/3214	77.3 ± 7.9	BMI	*aOR*	9
Garcia	2021	Spain	Cohort	90/299	82.3 ± 8.0	age, origin home, outdoor life, walking, immobility, nutritional risk	*aOR*	9
Gazineo	2019	Italy	Cohort	91/761	83.68 ± 7.9	Braden score, diaper, catheter, foam valve, surgical procedure	*aHR*	7
Han	2018	Korea	Cohort	1368/34,287	≥ 65	female, age, admission via wheelchair, drowsy, Braden score	*aOR*	7
Jaul	2019	Israel	Cross-sectional	23/40	median 73.5	dementia, spasticity	*aOR*	9
Kartal	2023	Turkey	Cross-sectional	117/382	76.20 + 8.36	age, BMI, clinical diagnosis, length of stay, admission via stretcher-wheelchair, tube feeding, insulin and steroids	*RR*	8
Man	2013	China	Cohort	17/229	83.35 ± 7.69	hypotension, use of restraint, length of stay	*aOR*	8
Mecocci	2005	Italy	Cohort	412/1379	78.38 ± 7.2	cognitive impairment	*aOR*	9
Mohammed	2024	Egypt	Cohort	31/334	72.35 ± 8.1	nutritional risk	*aOR*	8
Moon	2021	Korea	Cohort	328/8263	73.84 ± 6.33	age, admission via emergency department, low income, incontinence, immobility	*aOR*	9
Ottaviani	2024	Italy	Cohort	152/316	85 ± 7	age, mobility, friction/shear, albumin, hemoglobin, Barthel, Mini Nutritional Assessment	*aOR*	8
Papanikolaou	2002	England	Cohort	47/213	≥ 65	poor appetite, broken skin, incontinence, age, cancer, Parkinson	*β*	8
Papier	2022	Israel	Cohort	80/895	77.6 ± 9.1	Norton score, albumin, low intake	*aOR*	7
Rademakers	2007	Netherlands	Cohort	214/722	76.9 to 87.5	diabetes, urinary tract infection, hip dislocation, American Society of Anesthesiologists Classification, time to surgery	*aOR*	8
Stephenson	2023	Poland	Cross-sectional	143/2122	82.18 ± 7.90	nutritional risk	*aOR*	10

“USA” United States of America. “Scores” represents the scores of studies quality evaluation

### Quality assessment

The quality of the studies was assessed using the AHRQ checklist or the NOS, with scores ranging from 7 to 10, indicating generally moderate to high methodological quality (Table 1). This evaluation supports the reliability of the findings presented in our meta-analysis.

### Prevalence of pressure injuries

A total of 70,340 participants were included in the analysis. Among them, 5792 participants developed PI during hospitalization, corresponding to an overall prevalence of approximately 8.2%.

### Results of Meta-analysis

We performed risk factors that were reported in two or more studies. A total of 10 risk factors were included. For each factor, both fixed-effect and random-effects models were applied. Specifically, factors with high heterogeneity were analyzed using random-effects models, while those with low heterogeneity were analyzed using fixed-effect models. Table 2 summarizes the meta-analysis results of two models. Finally, a total of 8 risk factors were found to be statistically significant. For interpretability, the identified risk factors were further classified into two categories: non-modifiable patient characteristics and modifiable care-related factors.

**Table 2:** Meta-analysis results of risk factors of PI in elderly inpatients

Risk Factors	*N*	Random Effect Model				Fixed Effect Model		
		OR	CI	*P*-val	I^2^	OR	CI	*P*-val
age	9	1.06	(1.03, 1.09)	0.0001	83.70	1.04	(1.03, 1.05)	< 0.0001
immobility	4	4.54	(3.09, 6.67)	0.0000	74.20	4.45	(3.69, 5.37)	< 0.0001
albumin	3	1.12	(0.72, 1.74)	0.6250	85.70	1.17	(1.12, 1.22)	< 0.0001
incontinence	3	9.97	(2.33, 42.75)	0.0020	87.20	4.00	(2.75, 5.82)	< 0.0001
nutritional risk	3	3.00	(1.78, 5.05)	0.0000	80.20	2.41	(1.99, 2.92)	< 0.0001
BMI	2	0.39	(0.05, 3.34)	0.3910	78.30	0.94	(0.92, 0.96)	< 0.0001
diabetes	2	1.60	(1.21, 2.11)	0.0010	0.00	1.60	(1.21, 2.11)	< 0.0001
ICU stay	2	1.93	(1.46, 2.55)	0.0000	0.00	1.93	(1.46, 2.55)	< 0.0001
time to surgery	2	2.06	(1.58, 2.69)	0.0000	4.20	2.07	(1.60, 2.67)	< 0.0001
length of stay	2	1.05	(1.01, 1.09)	0.0146	65.00	1.05	(1.02, 1.07)	< 0.0001

Notes: “N” means the number of studies

**Fig. 2:**
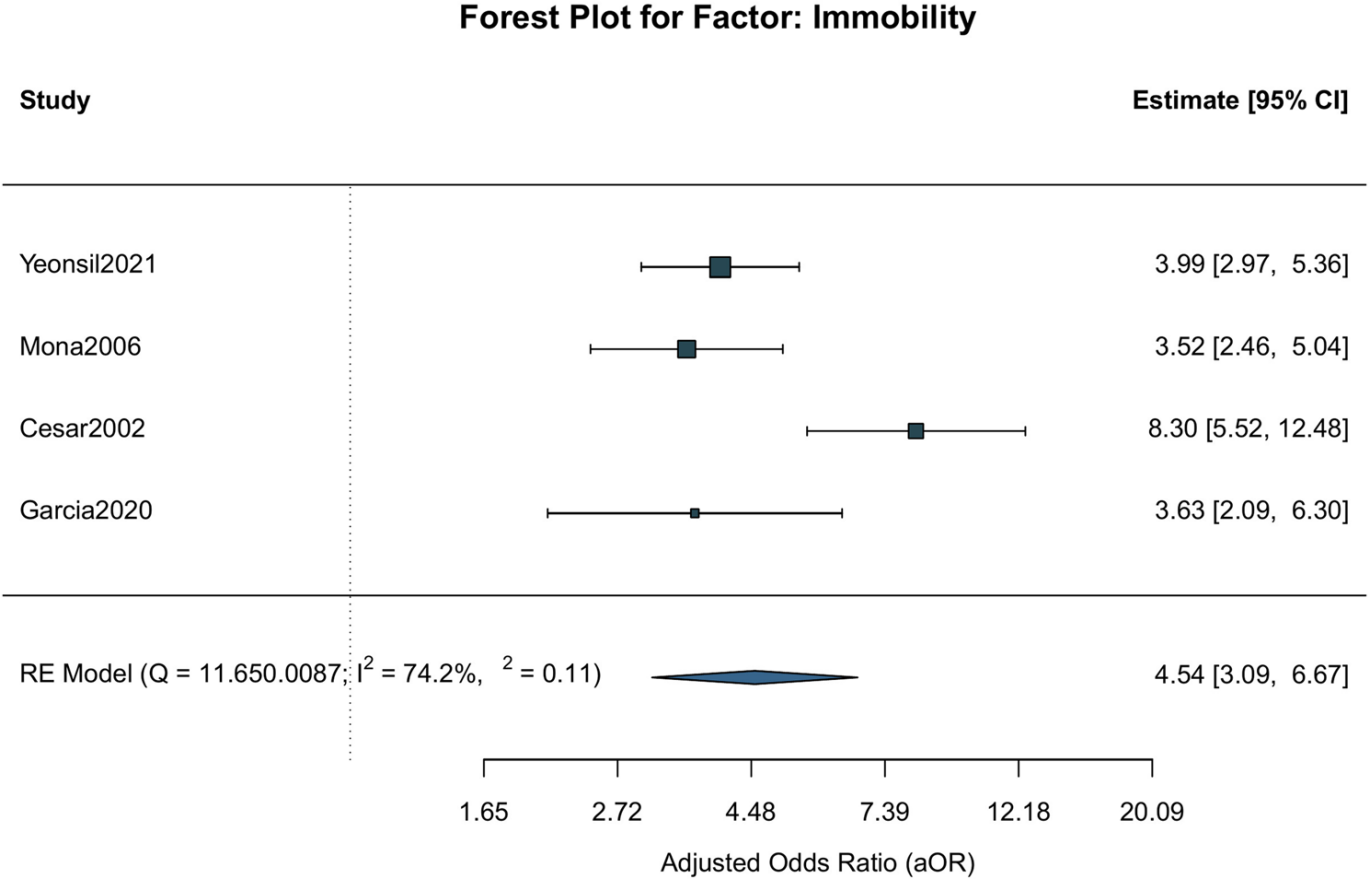
Forest plot of immobility

The results of the meta-analysis showed that risk factors of non-modifiable patient characteristics: age (*OR* = 1.06, 95%*CI*: 1.03 to 1.09, *P* = 0.0001), immobility (*OR* = 4.54, 95%*CI*: 3.09 to 6.67, *P* < 0.0001), diabetes (*OR* = 1.60, 95%*CI*: 1.21 to 2.11, *P* = 0.0010) and durations of prior ICU stay (*OR* = 1.93, 95%*CI*: 1.46 to 2.55, *P* < 0.0001); risk factors of modifiable care-related factors: incontinence (*OR* = 4.54, 95%*CI*: 2.33 to 42.75, *P* = 0.0020), nutritional risk (*OR* = 3.00, 95%*CI*: 1.78 to 5.05, *P* = 0.0020), time from admission to surgery (*OR* = 2.07, 95%*CI*: 1.60 to 2.67, *P* < 0.0001) and length of stay (*OR* = 1.05, 95%*CI*: 1.01 to 1.01, *P* = 0.0146). Those are statistically significant associated with the occurrence of PI in elderly inpatients. In contrast, albumin (*OR* = 1.12, 95%*CI*: 0.72 to 1.74, *P* = 0.6250) and BMI (*OR* = 0.39, 95%*CI*: 0.05 to 3.34, *P* = 0.3910) were not significantly associated. The forest plot as an example of the relationship between comorbid immobility and the occurrence of PI in elderly inpatients (Fig. 2).

### Sensitivity analyses

To assess the stability of the meta-analysis results, sensitivity analyses were performed comparing the random-effects model and the fixed-effects model (Table 2). Excepting incontinence, the magnitude of the effect estimates differed slightly, the direction and statistical significance remained consistent across models, indicating the findings are stable. Factor BMI and albumin yielded a statistically significant association in the fixed-effects model, while not in the random-effects model.

### Publication bias assessment

Due to the limited number of included studies, we did not perform publication bias assessments such as funnel plots or Egger’s test.

### Narrative synthesis

In addition, several studies reported effect estimates such as aRR, aHR and RR. They were not included in the meta-analysis but are summarized in Table 3.

**Table 3:** Studies with effect estimates not pooled in the meta-analysis

Study	Factors	Effect estimates	Values	95%CI
Baumgarten2012 [20]	length of stay in emergency department	aRR	0.680	(0.48, 0.96)
	time from admission to surgery		1.620	(1.24, 2.11)
	general anesthesia		0.660	(0.49, 0.88)
Gazineo2019 [29]	Braden score	aHR	0.884	(0.806, 0.969)
	diaper		1.007	(1.001, 1.013)
	catheter		1.013	(1.006,1.019)
	foam valve		1.017	(1.01, 1.023)
	osteosynthesis		1.876	(1.183, 2.975)
Kartal2023 [35]	age	RR	2.551	(1.616, 4.026)
	BMI		0.842	(0.786, 0.942)
	clinical diagnosis		1.647	(1.055, 2.569)
	length of stay		10.681	(6.444, 17.704)
	non-ambulatory admission		3.947	(2.009, 7.753)
	tube feeding		1.380	(1.212, 1.572)
	antibiotics and insulins		6.986	(2.730, 17.875)

Baumgarten et al. (2012), [20] a cohort study of 658 elderly patients, found that time from admission to surgery > 24 h (aRR = 1.62, 95%*CI*: 1.24 to 2.11) was associated with increased hospital-acquired PI risk, while length of stay in emergency department > 6 h (aRR = 0.68, 95%*CI*: 0.48 to 0.96) and general anesthesia (aRR = 0.66, 95%*CI*: 0.49 to 0.88) were associated with reduced risk.

Gazine et al. (2019) [29] identified risk factors associated with hospital-acquired PI using multivariate Cox regression analysis. Several factors were found to be significant. Specifically, a higher Braden score was associated with a lower risk (aHR: 0.884, 95% CI: 0.806 to 0.969). Conversely, factors such as diapers postoperatively (aHR: 1.007, 95% CI: 1.001 to 1.013), urinary catheters (aHR: 1.013, 95% CI: 1.006 to 1.019), increased percentage of days using foam valves preoperatively (aHR: 1.017, 95% CI: 1.010 to 1.023), and undergoing osteosynthesis (aHR: 1.876, 95% CI: 1.183 to 2.975) were positive and significant.

Kartal et al. (2023),[35] a cross-sectional analysis of 382 patients, showed that age ≥ 75 years (RR = 2.551, 95% CI: 1.616 to 4.026), BMI (RR = 0.842, 95% CI: 0.786 to 0.942), clinical diagnoses related to cardiovascular, respiratory, and COVID-19 systems (RR = 1.647, 95% CI: 1.055 to 2.569), length of stay ≥ 15 days (RR = 10.681, 95% CI: 6.444 to 17.704), non-ambulatory admission (RR = 3.947, 95% CI: 2.009 to 7.753), tube feeding (RR = 1.380, 95% CI: 1.212 to 1.572), and treatment with antibiotics and insulins (RR = 6.986, 95% CI: 2.730 to 17.875) were all significantly associated with increased PI risk.

## Discussion

Age, diabetes, and duration of prior ICU stay represent intrinsic patient vulnerabilities that are not amenable to modification. These factors reflect a higher degree of physiological frailty, impaired tissue perfusion, and chronic metabolic compromise, all of which predispose elderly individuals to PI development. For example, age-related skin changes include loss of dermal vessels, thinning of the epidermis, flattening of the dermal-epidermal junction, loss of elastic fibers, loss of subcutaneous fat, decreased rate of epithelialization, and decreased cell viability [37]. Persistent elevation of blood glucose induces metabolic dysregulations that result in peripheral neuropathy and vasculopathy. The ensuing loss of protective sensation, combined with compromised microcirculation (due to endothelial damage and capillary basement membrane thickening) and impaired immunity, significantly elevates susceptibility to PI[38]. It is also one of the main reasons why wounds are difficult to heal [39]. Longer durations of prior ICU stay may be associated with sustained immobility, hypo-perfusion, and exposure to risk-enhancing interventions (e.g., mechanical ventilation[40], vasopressors [41]), all of which may exacerbate risk of PI.

In contrast, immobility, incontinence, nutritional risk, time from admission to surgery and length of stay are dynamic, extrinsic factors that are closely tied to the quality and continuity of nursing delivered during hospitalization.

Prolonged immobility prevents natural pressure relief. Unrelieved pressure between bony prominences (like sacrum, hip, heel) and support surfaces compresses small blood vessels [42]. Inadequate blood flow deprives tissue of oxygen and nutrients, causing a buildup of metabolic waste, leading to local cell death and tissue necrosis [42]. Similarly, the Clinical Practice Guideline 2025 states that repositioning should be performed every two to three hours for at-risk individuals, particularly those with limited mobility [1]. 

Poor nutrition impairs skin integrity and wound healing. Protein deficiency impairs fibroblast proliferation, collagen synthesis, angiogenesis, and immune function, weakening wound structure [43]. Inadequate calories force the body to break down protein for energy, leading to further muscle and structural protein loss [44]. Lack of micronutrients impaired enzymatic reactions essential for tissue regeneration and immunity [45]. The clinical practice also showed that malnourished patients receiving intensive nutrition had better PI healing outcomes than standard care [46]. We recommend nutritional screening to enhance individualized nutritional care for patients to decrease PI.

Incontinence (urinary and fecal) showed the largest cumulative effect size in our analyses, but the confidence interval was extremely wide (*OR* = 9.97, 95% CI: 2.33–42.75). This was mainly driven by the study [25] that reported an unusually high OR, which substantially influenced the pooled estimate. Overall, Patients with incontinence are 4.5 times more likely to develop PI than those without incontinence. Incontinence exposes the skin to prolonged moisture and chemical irritants. Urine, often alkaline due to ammonia, raises skin pH, compromising the acid mantle that protects against pathogens; Feces contain digestive enzymes (e.g. proteases, lipases) that degrade lipids and proteins in the stratum corneum, further weakening the skin [47]. Moreover, other studies confirmed that dual incontinence was more damaging to skin integrity than urinary incontinence alone [48, 49]. This is consistent with the results of our meta-analysis. we recommend that clinicians strengthen the assessment of patients’ excretory function as part of routine PI risk screening. For patients with incontinence, particular attention should be paid to the condition of the perianal skin.

Prolonged time from admission to surgery is associated with a higher risk of PI, possibly due to extended periods of immobility and inadequate preventive care during the preoperative phase. Patients awaiting surgery may remain in a catabolic state, and delays in surgical intervention are often accompanied by increased exposure to risk factors such as malnutrition, hemodynamic instability, and limited mobility, which collectively increase susceptibility to tissue breakdown. Clinically, patients experiencing prolonged delays exhibited significantly higher odds of PI across various surgical cohorts [50, 51]. These findings suggest a dose–response relationship where longer delays incrementally increase ischemic exposure and pressure injury risk. So, reducing the time from admission to surgery is important, which calls for improved coordination among inpatient ward, surgical, and geriatric teams to streamline preoperative assessments and avoid unnecessary delays.

The length of stay is a common factor. It creates cumulative and synergistic effects — cumulative exposure to multiple risk factors, which we refer — significantly heightening the risk of PI.

BMI and albumin are not statistically associated with the occurrence of PI in elderly inpatients. It is an interesting finding because low BMI and hypoalbuminemia have traditionally been considered indicators of malnutrition. But their role as independent predictors remains controversial. Several recent studies [52, 53] have questioned the predictive value of albumin alone due to its sensitivity to acute illness and inflammation, rather than true nutritional status. Similarly, evidence [54] indicated a non-linear (J‑shaped/U‑shaped) association between BMI and PI: both underweight and obesity significantly increase risk compared to normal BMI. It is making pooled results inconsistent across studies.

Unlike the commonly recognized risk factors for PI — such as pressure, friction, shear forces, and moisture — the study identified several patient- and treatment-related factors, which may be particularly relevant in the elderly population. For instance, incontinence is a significant and modifiable risk factor in our analysis. Compared to younger individuals, older adults are more prone to fractures [55] and cardiovascular diseases [56]. These conditions are often accompanied by varying degrees of urinary or fecal incontinence, which can prolong tissue exposure to pressure and compromise the protective mechanisms of skin, increasing the risk of PI. Therefore, we recommend that clinicians perform comprehensive and individualized risk assessments for hospitalized elderly patients, focusing on physical function, excretion, comorbidities, and nutritional support. Such targeted assessments enable the early identification of high-risk individuals. Timely evidence-based prevention strategies and continuously precise nursing aimed at reducing PI.

The study has several limitations: (1) The risk factors analyzed varied widely among studies, and some cases could not be synthesized, thereby limiting the comprehensiveness of this meta-analysis. (2) Some risk factors were explored in only a few studies, reducing the statistical power and generalizability of the findings. (3) There was considerable variability in sample sizes across studies, which could contribute to heterogeneity and affect the stability of the pooled results.

## Conclusion

This meta-analysis identified the age, immobility, incontinence, nutritional risk and length of stay, time of prior ICU stay, time from admission to surgery, and diabetes as significant risk factors for PI in elderly inpatients. These findings provide more scientific, stable, and reliable evidence than individual studies, and may help clinical personnel recognize the high-risk patients and implement preventive strategies. However, because most included studies were retrospective, the findings may reflect associations influenced by confounding rather than definitive causal relationships. Future multicenter, large-sample, high-quality randomized controlled trials are needed to further validate these findings and strengthen causal inferences.

## Supplementary Information

Supplementary Material 1

## Data Availability

Data sharing is not applicable to this article as no datasets were generated or analysed during the current study.

## Abbreviations

aHR: Adjusted hazard ratios
aOR: Adjusted odds ratios
aRR: Adjusted risk ratios
CI: Confidence intervals
OR: Odds ratios
PI: Pressure injury
β: Regression coefficient
RR: Risk ratio
SEs: Standard errors
