# Supplementary material for: Risk factors of pressure injury in elderly inpatients: a systematic review and meta-analysis
Source: BMC Geriatr. 2025 Nov 10;25:874. doi: 10.1186/s12877-025-06517-0 (PMC12599044; doi:10.1186/s12877-025-06517-0)
Supplement: Supplementary file 1 — Supplementary Material 1 [file 12877_2025_6517_MOESM1_ESM.pdf]

2025-03-01

PubMed

Result: 377

#1 Aged[MeSH Terms] (3,650,163)

#2 Inpatients[MeSH Terms] (32,197）

#3 Pressure Ulcer[MeSH Terms] (14,712)

#4 Risk Factors[MeSH Terms] (1,025,519)

#5 "older adult*"[Title/Abstract] OR "older people"[Title/Abstract] OR "elderly"[Title/Abstract] OR "older population"[Title/Abstract] OR "senior citizen*"[Title/Abstract] (481,042)

#6 "hospitalized"[Title/Abstract] OR "hospitalization"[Title/Abstract] OR "inpatient*"[Title/Abstract] OR "in-hospital" (550,355)

#7 "bed sore*"[Title/Abstract] OR "bedsore*"[Title/Abstract] OR "pressure sore*"[Title/Abstract] OR "decubitus"[Title/Abstract] OR "pressure injury"[Title/Abstract] OR "skin breakdown"[Title/Abstract] (13,211)

#8 "related factor*"[Title/Abstract] OR "Determinant"[Title/Abstract] OR "Predicted factors"[Title/Abstract] OR "contributing factor*"[Title/Abstract] OR "influencing factor*"[Title/Abstract] OR "associated factor*"[Title/Abstract] OR "relevant factor*"[Title/Abstract] (288,804)

#9 #1 OR #5 (3,782,957)

#10 #2 OR #6 (556,333)

#11 #3 OR #7 (22,350)

#12 #4 OR #8 (1,287,711)

#13 #9 AND #10 AND #11 AND #12 (377)

Web of science (WOS)

Result: 1,738

#1 TS=(older adult* OR older people OR elderly OR older population OR senior citizen*) (1,983,766)

#2 TS=(hospitalized OR hospitalization OR inpatient* OR in-hospital) （1,004,527）

#3 TS=(pressure ulcer OR bed sore* OR bedsore* OR pressure sore* OR decubitus OR pressure injury OR skin breakdown) （312,749）

#4 TS=(risk factor* OR related factor* OR determinant OR predicted factors OR contributing factor* OR influencing factor* OR associated factor* OR relevant factor*) （8,569,848）

#5 #1 AND #2 AND #3 AND #4 （1,738）

Embase

Result: 622

#1 'aged'/exp （4,186,502）

#2 'hospital patient'/exp （253,475）

#3 'decubitus'/exp （29,385）

#4 'risk factor'/exp （1,515,824）

#5 'older adult*':ab,ti OR 'older people':ab,ti OR elderly:ab,ti OR 'older population':ab,ti OR 'senior citizen*':ab,ti (635,480)

#6 hospitalized:ab,ti OR hospitalization:ab,ti OR inpatient*:ab,ti OR 'in hospital':ab,ti (898,787)

#7 'pressure ulcer':ab,ti OR 'bed sore*':ab,ti OR bedsore*:ab,ti OR 'pressure sore*':ab,ti OR decubitus:ab,ti OR 'pressure injury':ab,ti OR 'skin breakdown':ab,ti (23,403)

#8 'risk factor*':ab,ti OR 'related factor*':ab,ti OR determinant:ab,ti OR 'predicted factors':ab,ti OR 'contributing factor*':ab,ti OR 'influencing factor*':ab,ti OR 'associated factor*':ab,ti OR 'relevant factor*':ab,ti (1,569,563)

#9 #1 OR #5 (4,338,563)

#10 #2 OR #6 (954,214)

#11 #3 OR #7 (37,802)

#12 #4 OR #8 (2,250,033)

#13 #9 AND #10 AND #11 AND #12 (622)

Cochrane Library

Result:465

#1 MeSH descriptor: [Aged] explode all trees (276,051)

#2 MeSH descriptor: [Inpatients] explode all trees (1,641)

#3 MeSH descriptor: [Pressure Ulcer] explode all trees (1,107)

#4 MeSH descriptor: [Risk Factors] explode all trees (37,632)

#5 (older adult* OR older people OR elderly OR older population OR senior citizen*):ti,ab,kw (110,950)

#6 (hospitalized OR hospitalization OR inpatient* OR in-hospital):ti,ab,kw (269,352)

#7 (pressure ulcer OR bed sore* OR bedsore* OR pressure sore* OR decubitus OR pressure injury OR skin breakdown):ti,ab,kw (17,767)

#8 (risk factor* OR related factor* OR determinant OR predicted factors OR contributing factor* OR influencing factor* OR associated factor* OR relevant factor*):ti,ab,kw (503,863)

#9 #1 OR #5 (350,991)

#10 #2 OR #6 (269,352)

#11 #3 OR #7 (17,767)

#12 #4 OR #8 (503,866)

#13 #9 AND #10 AND #11 AND #12 (465)

Nursing and Allied-Health Literature (CINAHL)

Result:427

S1 MH aged (938,912)

S2 TI (older adult* OR older people OR elderly OR older population OR senior citizen*) OR AB (older adult* OR older people OR elderly OR older population OR senior citizen*) (223,239)

S3 S1 OR S2 (1,005,597)

S4 MH inpatients (89,409)

S5 TI (hospitalized OR hospitalization OR inpatient* OR in-hospital) OR AB (hospitalized OR hospitalization OR inpatient* OR in-hospital) (211,625)

S6 S4 OR S5 (267,919)

S7 MH pressure ulcer (16,153)

S8 TI (pressure ulcer OR bed sore* OR bedsore* OR pressure sore* OR decubitus OR pressure injury OR skin breakdown) OR AB (pressure ulcer OR bed sore* OR bedsore* OR pressure sore* OR decubitus OR pressure injury OR skin breakdown) (16,980)

S9 S7 OR S8 (22,051)

S10 MH risk factors (193,473)

S11 TI (risk factor* OR related factor* OR determinant OR predicted factors OR contributing factor* OR influencing factor* OR associated factor* OR relevant factor*) OR AB (risk factor* OR related factor* OR determinant OR predicted factors OR contributing factor* OR influencing factor* OR associated factor* OR relevant factor*) (456,098)

S12 S10 OR S11 (573,842)

S13 S3 AND S6 AND S9 AND S12 (427)
